# Supplementary figures and images for: Exploring the variation in muscle response testing accuracy through repeatability and reproducibility
Source: PLoS One. 2025 Jul 8;20(7):e0326208. doi: 10.1371/journal.pone.0326208 (PMC12237029; doi:10.1371/journal.pone.0326208)

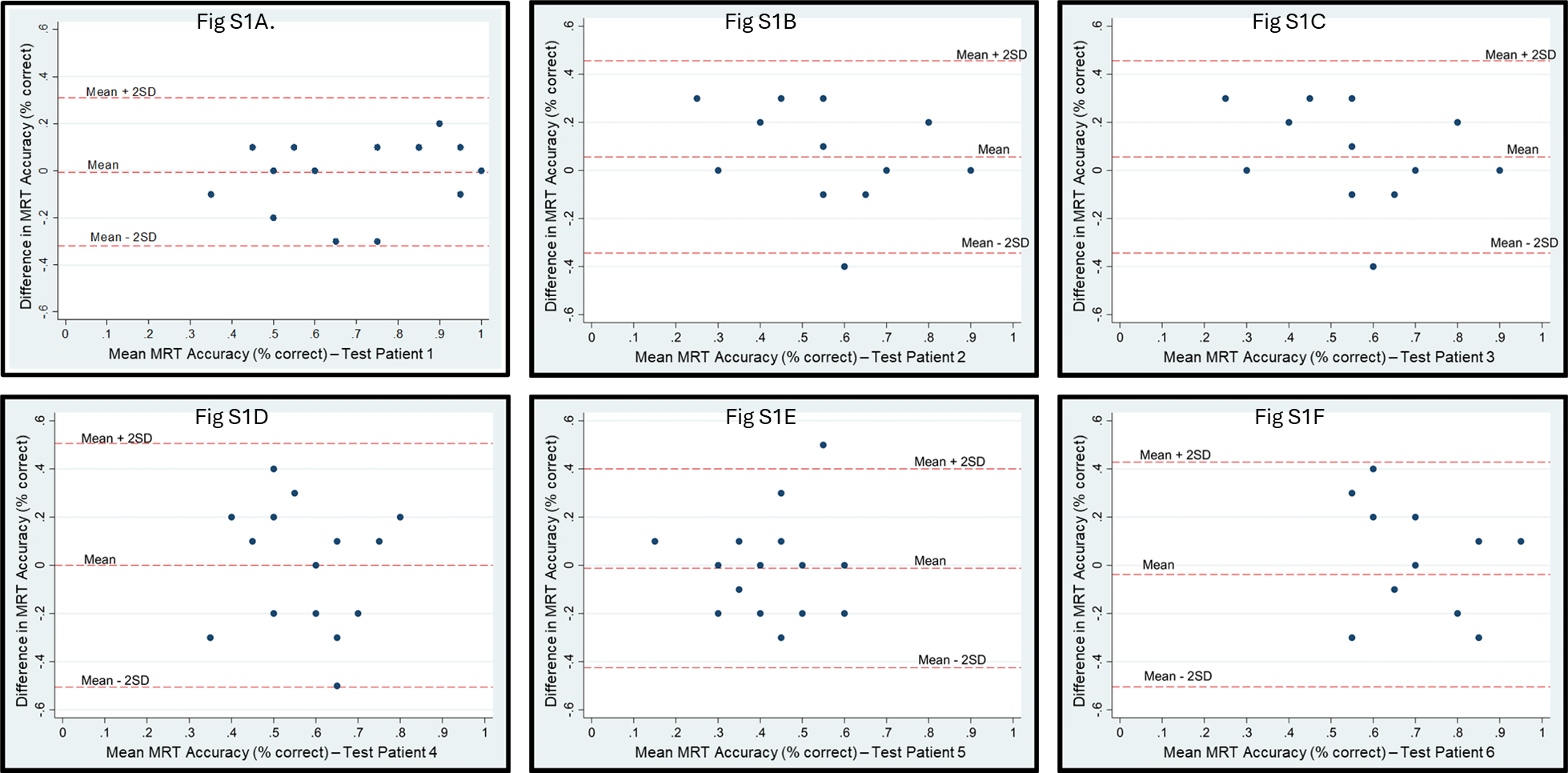

Supplement: S1 Fig — Bland-Altman Plots of the difference between block 1 and block 2 scores (y-axis) against mean score (x-axis). (TIF) [file pone.0326208.s001.tif]
